# A new reality for telehealth: A simulation-based comparison of wearable mixed reality with videoconferencing for clinician-to-clinician telehealth

**DOI:** 10.1177/20552076251388404

**Published:** 2025-11-03

**Authors:** Chiara Santomauro, Daniel Best, Beth Wray, Felicity Burgmann, Tricia Pilotto, Sarah Pearce, Mia McLanders

**Affiliations:** 1 Safety Science Innovation Lab, 5723 Griffith University, Nathan, Brisbane, Australia; 2 School of Psychology, 1974 The University of Queensland, St Lucia, Australia; 3 1288 Queensland Virtual Hospital, Clinical Excellence Queensland, Herston, Brisbane, Australia; 4 Clinical Skills Development Service, 157827 Metro North Health, Herston, Brisbane, Australia; 5 157827 Jamieson Trauma Institute, Metro North Health, Herston, Brisbane, Australia

**Keywords:** Mixed reality, HoloLens, simulation, telehealth, telemedicine, videoconferencing

## Abstract

**Introduction:**

Telehealth is crucial to the provision of high-quality treatment of critically unwell patients in rural areas. Our aim was to explore the benefits and limitations of a mixed reality (MR) headset for clinician-to-clinician telehealth in a simulated rural context, in which the advising clinician directly views the treating clinician's point-of-view and uses holographic annotations to provide visual guidance in three-dimensional space.

**Methods:**

Twenty-five clinicians trialled a MR headset—the Microsoft HoloLens 2—and evaluated it against a trolley-based videoconferencing device used in current practice. Each participant trialled the devices while role-playing one of two perspectives: the Rural Clinician accessing support or the Advising Clinician providing support.

**Results:**

Advising Clinicians had higher ratings of usability and self-efficacy, and lower ratings of mental workload, when providing support via the MR headset compared to the videoconferencing trolley (ps < 0.035). However, Rural Clinicians rated the MR headset lower on usability compared to the videoconferencing trolley (p = 0.020), and rated their self-efficacy and mental workload equally when using both devices (ps > 0.253). Participants generally preferred to use the MR headset over the videoconferencing trolley. On average, scenarios took 1 minute longer to complete when using the MR headset (p < 0.001).

**Discussion:**

Wearable MR technology has the potential to improve the quality of support provided to rural clinicians; however, it may be more beneficial for those dialling in to the device (advising clinicians) rather than those wearing the device (rural clinicians). Factors that may limit its safety and efficiency are discussed.

## Introduction

Telehealth—the delivery of healthcare services over distance, enabled by information and communication technologies—is crucial to the provision of high-quality patient care in rural and remote areas. The use of telehealth during rural and remote care consistently demonstrates positive outcomes for both rural patients and clinicians by overcoming geographical barriers to accessing specialist advice and support.^1–4^

Videoconferencing is the most commonly used form of telehealth, but is limited in functionality. Moving toward more advanced telehealth models of care that require precision advice, greater collaboration, and healthcare outside the traditional hospital setting, comes the need for new features and technologies. Emerging mixed reality (MR) technologies offer a progression over standard videoconferencing by allowing the advising clinician to annotate, measure, manipulate, and point in a videoconference to better assist the rural clinician through procedures.^5^ Pairing MR software with a head-worn device ensures that the most appropriate camera angles of the patient are transmitted to the advising specialist, and that the visual guidance can be viewed by the treating clinician without averting their gaze away from the patient.^6^

Despite potential benefits, the use of head-worn displays in clinical contexts may elicit unintended consequences. For example, some evidence suggests that head-worn displays can worsen a wearer's performance on their primary task, even when the display is not being used at the time.^7^ Head-worn displays can also obstruct the field-of-view of the primary task, causing distraction, increasing cognitive load, and impairing situation awareness.^8–10^ Barriers that limit the successful implementation of telehealth (e.g. costs and technical competency of staff^11^) may be exacerbated by the introduction of technologies that are not widely used in healthcare, such as MR software and wearable cameras.

The aim of this study was to explore the benefits and potential limitations of a novel technology for clinician-to-clinician telehealth: a MR headset that may offer further point-of-care support to rural clinicians using holographic annotations in three-dimensional (3D) space. This aim was achieved through a simulation-based usability study—an evaluation technique that involves representative users trialling a product or service to highlight any issues or challenges associated with the product in practice^12^—in which clinicians trialled a MR headset for telehealth and evaluated it against a videoconferencing device used in current practice. This study was conducted as part of a program of research designed to improve the quality of telehealth support provided to rural and remote clinicians.

## Method

### Design

This was a mixed-design simulation study where all participants trialled two telehealth devices (a MR headset and a standard videoconferencing device), but only trialled these devices from one of two conditions representing the dual perspectives of a clinician-to-clinician telehealth consultation during rural care. Depending on their prior experience, participants were matched to either condition (a) the Rural Clinician accessing support, or condition (b) the Advising Clinician providing support. To ensure a standardised simulation experience for all participants, one clinician participated at a time and a researcher played the other role (either Advising Clinician or Rural Clinician). Each participant completed two blocks of the same four scenarios, trialling one telehealth device in each block. The order in which participants trialled each device was randomised and counterbalanced to minimise biases such as learning effects.

### Participants and recruitment

Participants were drawn from a wide-ranging convenience sample of medical, nursing, and allied health staff who were recruited via email advertisements and word-of-mouth on a volunteer basis with no additional exclusion criteria. Participants tended to have experience using telehealth although it was not a requirement to participate. In usability testing, a sample of 10 to 12 participants per condition is considered a sensible baseline range^13^; thus, 25 participants were recruited, with 13 participants in condition (a) and 12 participants in condition (b). Recruitment commenced in December 2023 and data collection occurred between January and May 2024. Participant demographics are presented in Table 1. For simplicity, participants in condition (a) will be referred to as Rural Clinicians and participants in condition (b) will be referred to as Advising Clinicians.

**Table 1.** Participant demographics.

Demographic	Rural Clinicians (n = 13)	Advising Clinicians (n = 12)
Sex		
Female	10	9
Male	3	3
Age (years)		
25–34	0	1
35–44	7	4
45–54	1	5
55–64	5	1
>65	0	1
Role		
Senior medical officer	0	5
Nurse educator	4	0
Nurse practitioner	1	2
Clinical nurse	2	0
Clinical director	1	1
Physiotherapist	1	1
Speech pathologist	1	1
Nursing director	1	0
Clinical lead	1	0
Clinical implementation support	1	01
Specialist	0	1
Principal advisor – virtual health sleep scientist	0	1
Clinical area (note: some participants work in more than one area)		
Emergency	4	3
Telehealth	1	5
Education and training	4	0
General practice (wound care)	0	1
Infectious diseases/sexual health	1	0
Maternity	1	0
Neonatology	0	2
Nursing	1	0
Physiotherapy	1	1
Speech pathology	1	1
Thoracic medicine	0	1
Years of experience	M = 22.9	M = 21.5
	SD = 12.9	SD = 12.2
Glasses		
Yes	8	7
No	5	5
Hearing aid		
Yes	0	0
No	13	12

Values are number of participants, except for experience reported in mean and standard deviation (SD) years.

### Ethics approval

This study was granted ethical approval by the Human Research Ethics Committees of the Royal Brisbane and Women's Hospital and Griffith University (HREC/2020/QRBW/62878 and 2020/631, respectively). Prospective participants were given an information sheet and provided written informed consent prior to scheduling a simulation session.

### Simulated environment and materials

**Figure 1.**
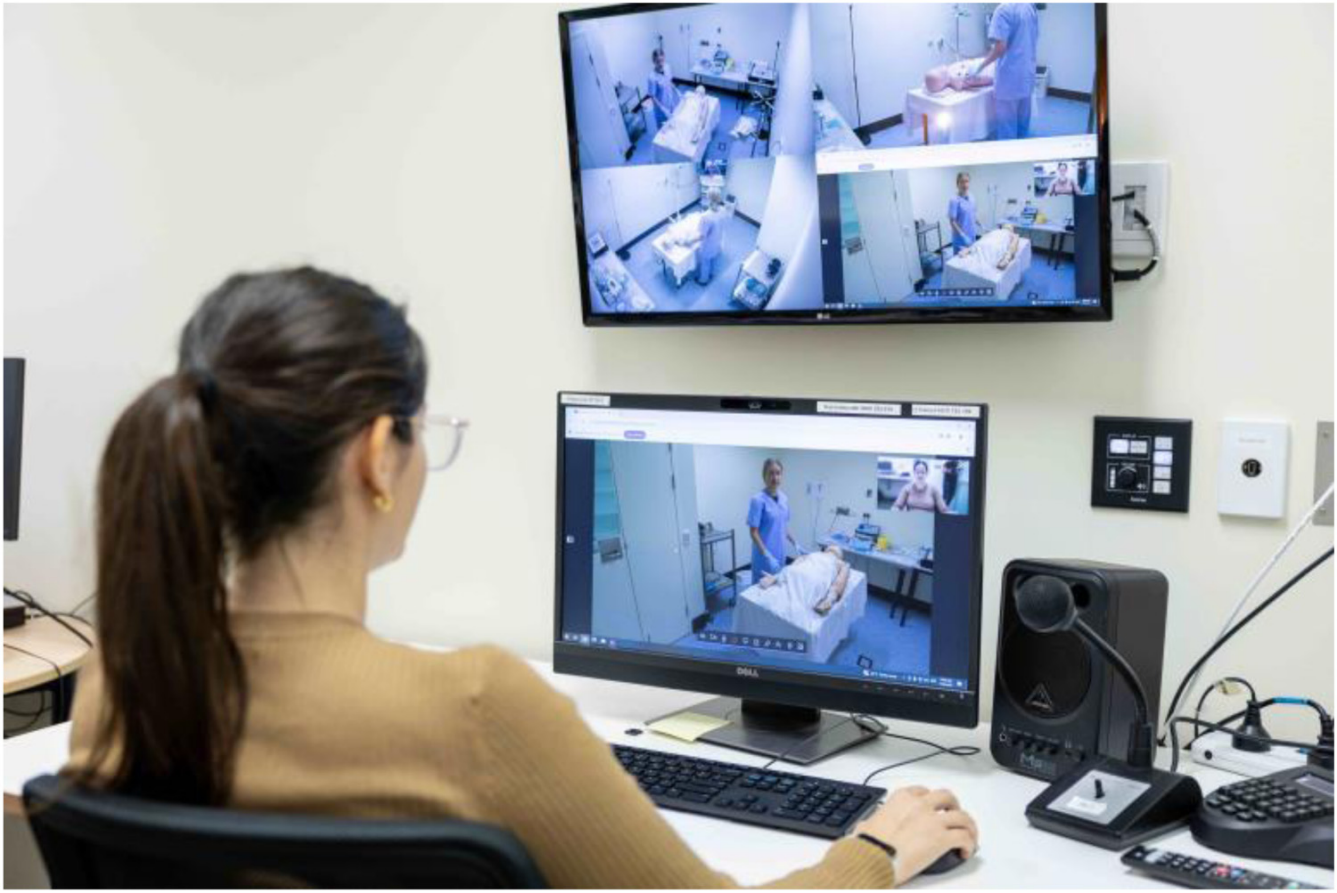
The Advising Clinician station.

Testing sessions were conducted in a simulation lab at the Clinical Skills Development Service located on the Royal Brisbane and Women's Hospital campus. The simulation lab was set up to mimic a rural patient bay, containing a manikin, equipment required for the scenarios, and an existing telehealth device. An Advising Clinician station was set up in an adjacent room with a desk and computer with an inbuilt webcam (Figure 1).

**Figure 2.**
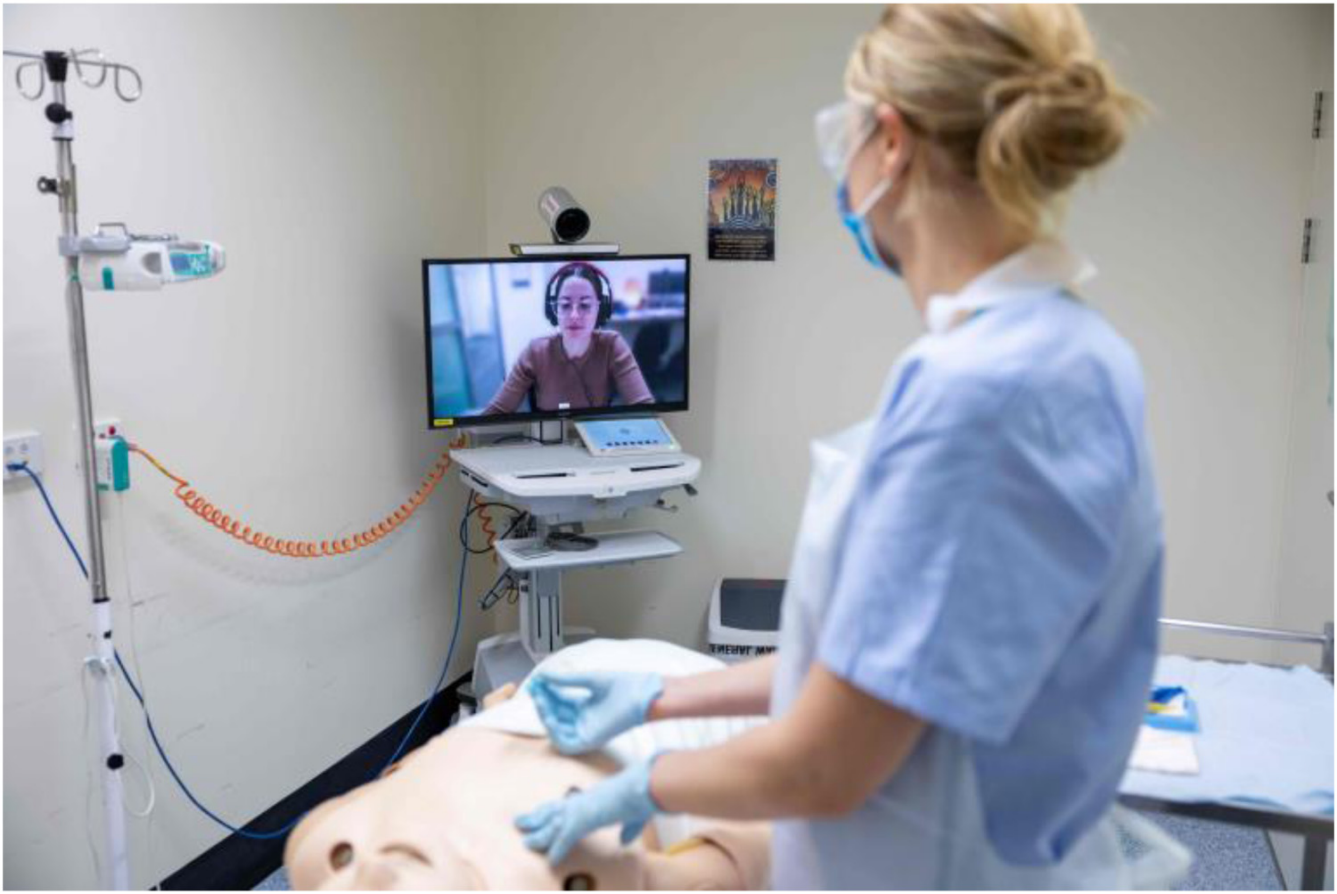
A Rural Clinician using the videoconferencing trolley.

The existing telehealth device was a trolley-based videoconferencing system which includes a camera, screen, and touch screen tablet (Figure 2). This device and similar variations are used solely for telehealth consultations and are available in over 200 facilities across the state of Queensland, Australia. Telehealth calls are made to the device through a web-based telehealth portal. Once a two-way video call is connected, the Advising Clinician can control the camera remotely with pan tilt zoom functions. The Rural Clinician can also wheel the device around the room to provide optimal views for the Advising Clinician.

**Figure 3.**
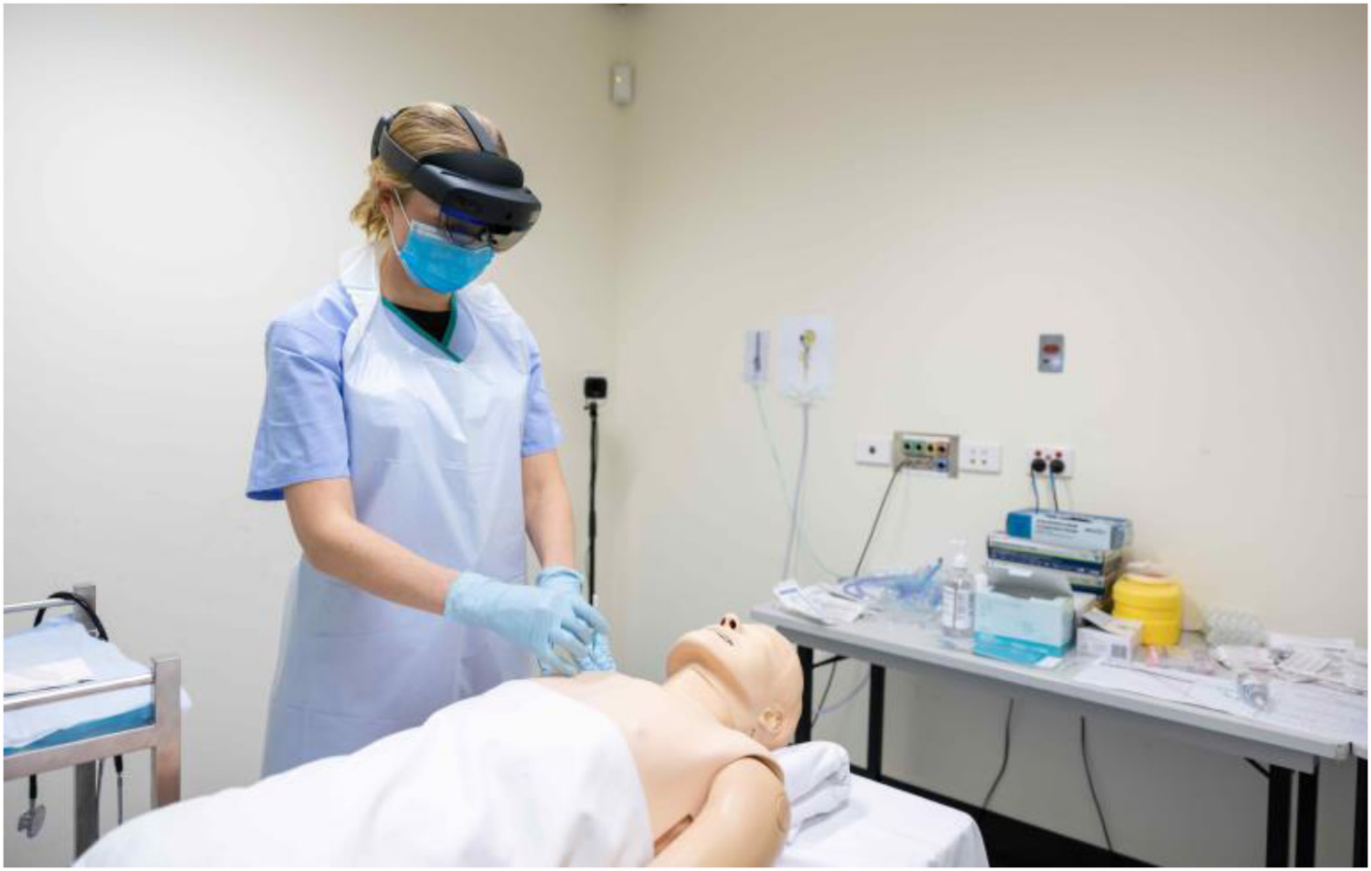
A Rural Clinician using the HoloLens 2.

The comparison device chosen for this study was the Microsoft (Redmond, WA) HoloLens 2, which is a head-worn fully self-contained holographic computer enabling MR functionality through a see-through lens (Figure 3). The headset runs on a Windows operating system and uses advanced optical projection to generate multidimensional full-colour holograms, so that the display can be pinned or anchored to the environment around the wearer. The headset features built-in sensors to enable a gaze-controlled cursor which is activated with gestures and/or voice commands. For this study, Microsoft Teams software was used with a Remote Assist add-on, enabling the sharing of annotations in 3D space while in a two-way video call. These annotations remain fixed to their specific locations in 3D space, even when the wearer moves around. This device provides additional functionality over existing telehealth devices by providing a point-of-view camera angle allowing hands-free guidance and additional portability and mobility for the Rural Clinician wearing the device. The HoloLens 2 also allows the Advising Clinician to annotate, drop arrows, and display reference material in the Rural Clinician's vision to enable greater collaboration during procedures, none of which is available with the existing telehealth devices.

For real-world clinical use, any rural clinician who might use the HoloLens 2 in a clinical situation would be required to work through a brief, one-off setup and calibration process to operate the HoloLens 2. This process includes logging into their Microsoft account, setting up a quick-access PIN number, opting in for an iris scan if desired (also for quick login access), and completing a short tutorial on the device's basic operation. Once this process is complete, staff have easy access to either make or receive Microsoft Teams calls via the HoloLens 2 with all the associated functionalities.

**Figure 4.**
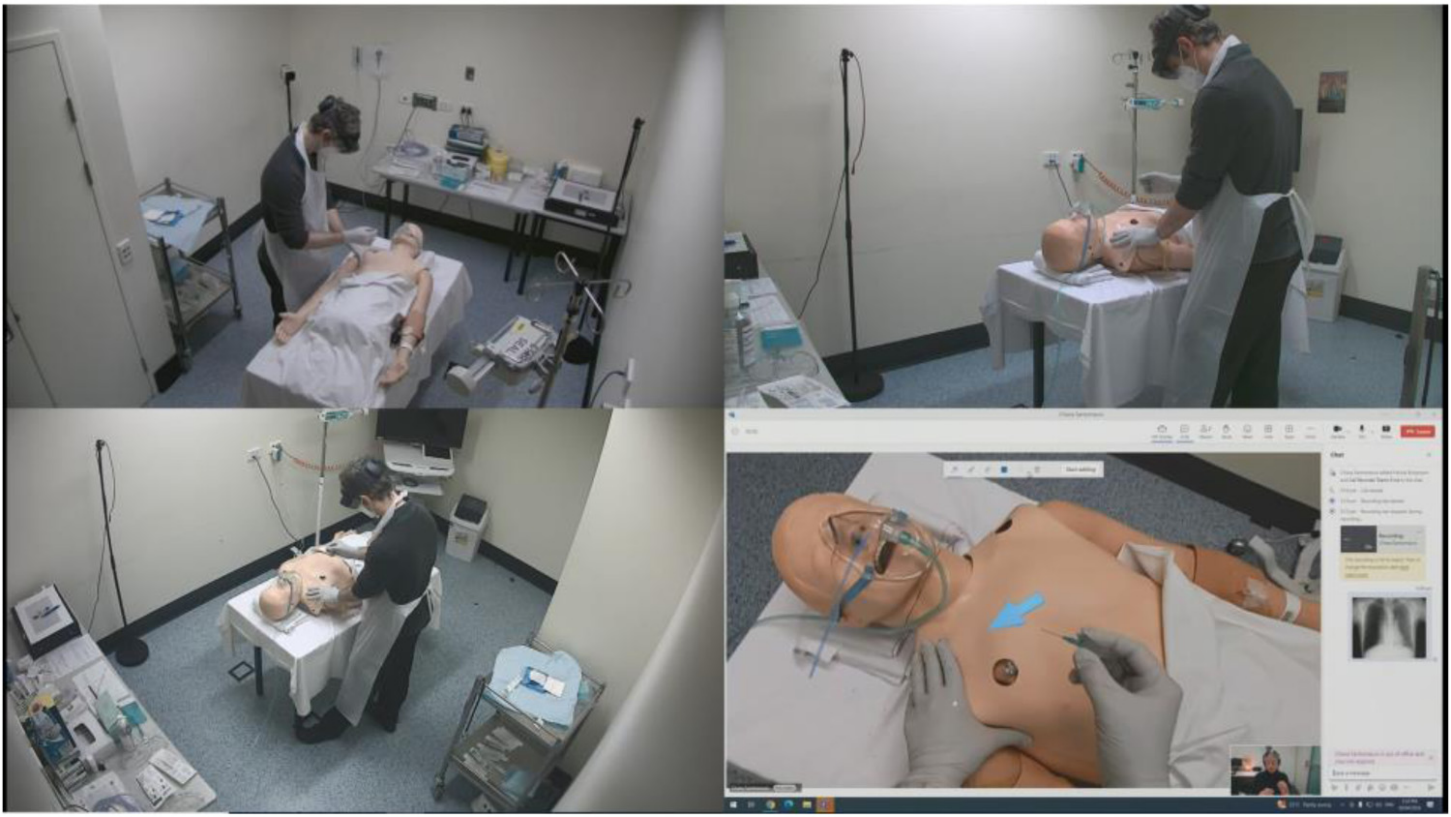
The four video sources used to capture usability evaluation data. The participant acting in the Rural Clinician role is shown in the top two and bottom left frames. The Advising Clinician's (played by the researcher) computer screen is shown in the bottom right frame, having just annotated an arrow on the manikin's chest.

Testing sessions were recorded using three cameras and a ceiling-mounted microphone in the simulated rural patient bay. Computer screen recordings from the Advising Clinician station were recorded in tandem, meaning that the four video sources were simultaneously recorded in a single view (Figure 4). These video sources were also displayed on a screen above the Advising Clinician station (Figure 1), providing a birds-eye-view of the room. This is similar to the state's rural telehealth infrastructure, with most rural emergency bays fitted with ceiling-mounted cameras used for videoconferencing.

### Measures

The objective of this study was to compare each device on outcomes that correlate with task performance (device usability,^14^ self-efficacy,^15^ and mental workload^14,16^). To measure subjective feedback from participants, a questionnaire was administered via the Qualtrics online survey platform (Provo, UT). Video recordings were used to capture scenario completion time. The primary outcomes measures were:

1. Device usability, adapted from the validated Post-Study System Usability Questionnaire (PSSUQ).^17^ This questionnaire assesses perceived device ease of use, functionality, and usefulness, with item wording and number of items tailored to each telehealth device and user perspective.
2. Self-efficacy, adapted from the validated New General Self-Efficacy Scale.^18^ This 8-item scale assesses the belief in one's ability to succeed in the future.
3. Mental workload, using the validated National Aeronautics and Space Administration Task Load Index (NASA-TLX).^19^ The eight NASA-TLX items measure subjective ratings of mental, physical, and temporal demand, performance (reverse-scored), effort, and frustration.

The secondary outcome measures were:

1. Device preference for each scenario.
2. Scenario completion time.
3. Perceived value of HoloLens 2 key functions.
4. Qualitative feedback on HoloLens 2. Specifically, one open-ended question for each of the following: usability, comfort, visual and audio quality, likes, dislikes, sickness symptoms, and clinical cases the device would be useful/not useful for.
5. Perceptions of scenario fidelity and realism.
6. Demographics.

### Simulated scenarios

Four simulated scenarios of rural patient care were developed in consultation with non-participant clinical colleagues (Table 2). The scenarios were deliberately designed to vary in properties such as urgency, acuity, and duration to elicit feedback on the different kinds of use cases that may be suitable for a wearable MR telehealth device.

**Table 2.** Scenario descriptions and corresponding functions used with each telehealth device.

Scenario description	Videoconferencing trolley	HoloLens 2
1. A patient has presented to a rural facility with extreme pain after an injury. The Rural Clinician is working alone and wants to request a schedule 8 drug order and check via telehealth	Videoconferencing	POV videoconferencing
2. A junior clinician working in a rural hospital has a patient who requires a frusemide infusion. They need help setting up a syringe driver, but no one is available in their facility. They are requesting assistance via telehealth to set up the syringe driver	Videoconferencing	POV videoconferencing
3. A Rural Clinician is treating a patient with breathing difficulties and oxygen saturations of 89% despite receiving oxygen through a Hudson mask. The clinician is unsure what to do next and is requesting telehealth assessment and support. The patient will require insertion of a nasopharyngeal airway	Videoconferencing	POV videoconferencing Annotation (on patient's head)
4. A Rural Clinician is treating a patient with a suspected collapsed lung. The clinician has listened to the chest and hears diminished breath sounds on the right side, and slight crackles on the left side. The patient has undergone a chest X-ray, but the clinician is unsure what to do next and is requesting telehealth support. The patient will require a needle decompression to reinflate the right lung	Videoconferencing	POV videoconferencing File sharing (chest X-ray) Annotation (on chest X-ray and patient's chest)

POV:  point-of-view.

### Procedure

**Figure 5.**
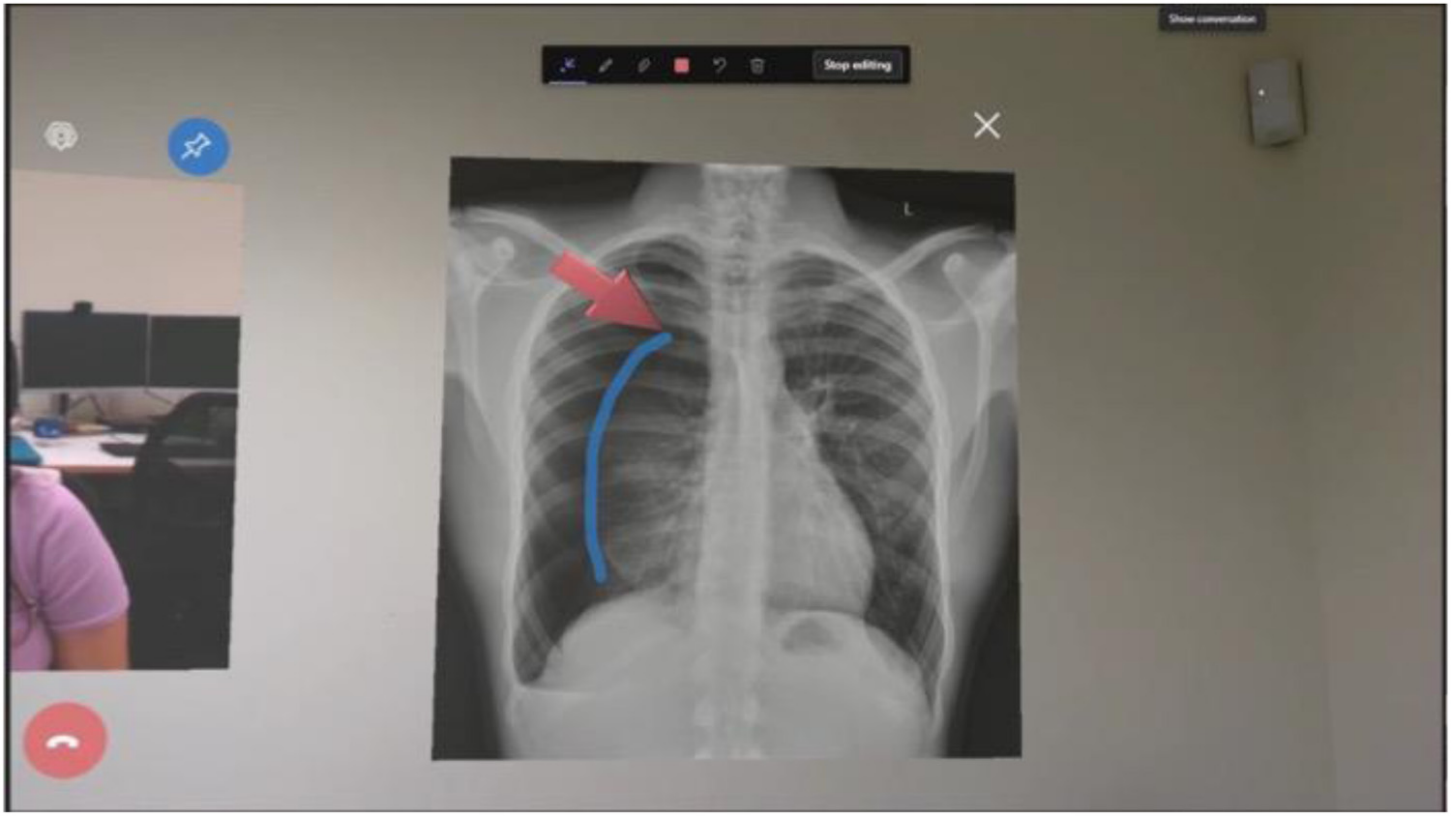
An example of file sharing and annotation used in scenario 4 with the HoloLens 2. The Advising Clinician shared the patient's chest X-ray with the Rural Clinician which appears in three-dimensional space beside the video call which has been pinned to a blank wall. The Advising Clinician has then annotated on the X-ray to educate the Rural Clinician on the pneumothorax and planned location for the needle decompression.

Upon arrival, each participant read and signed an information sheet and consent form explaining the details of the study and informing them that visual and audio footage of the scenarios will be recorded. The participant was then given an overview of the process, orientated to the simulation space, and instructed on how to operate the first device (for those acting in the Rural Clinician role) or software (for those acting in the Advising Clinician role). To ensure that the video call did not obstruct the Rural Clinicians’ view of the patient when using the HoloLens 2, part of the training involved learning how to pin video calls to a fixed location in 3D space. Participants were instructed to pin the video call to a blank wall, mimicking a physical computer screen mounted to the wall. Once the participant was confident to operate the device or software, the first block of scenarios began. Participants acting in the Rural Clinician role would remain in the simulated rural patient bay for the duration of the block, and the researcher would enter the room before each scenario to provide a brief context. The same process applied to participants acting in the Advising Clinician role, except that they were stationed in the adjacent room. Whoever was acting in the advising role (either the participant or the researcher) provided guidance via the first telehealth device and software, following detailed scripts to ensure standardisation of communication and tasks. When the HoloLens 2 was used, the scripts also included annotation and file sharing instructions (Figure 5). After the first block of scenarios, the participant completed the questionnaires for the first device. The above steps were then repeated for the second device in the second block. At the conclusion of both blocks, participants completed the remaining questionnaires including device preferences, scenario fidelity, and demographics. Participants were able to take breaks in between scenarios if desired, and all participants received a brief break after the first block while the researcher set up for the second block. Overall, each testing session lasted approximately 1.5 to 2 hours, dependent upon the level of training each participant required to feel confident operating the devices or software, and their pace working through the scenarios.

### Data analysis

All data were exported into Microsoft Excel worksheets. Statistical analyses were performed on quantitative data with open-access statistical software Jamovi (v2.3.28.0, Sydney, Australia), with two-tailed tests and an α level of .05. Data were analysed separately according to participant condition (Rural Clinician or Advising Clinician). Repeated-measures t-tests were used to compare devices. Average scores on each outcome measure were used in the analyses (rather than individual items). It is acceptable practice to use parametric tests on Likert scales because their composite scores are found to be approximate to interval-level measurement.^20,21^ Since parametric tests are more powerful than non-parametric tests, parametric tests were conducted for all variables unless specified where statistical assumptions were not met. When device comparisons were not possible, descriptive statistics are presented. There were no missing data and no participants were excluded from analyses. Qualitative data from open-ended survey items were summarised by grouping feedback into high-level categories based on the questions asked and frequency of responses.

## Results

### Primary outcomes

Table 3 presents the average scores on the main outcome variables: usability, self-efficacy, mental workload, and associated *p*-values to indicate statistically significant differences across devices.

**Table 3.** Descriptive statistics for primary outcomes across the two telehealth devices.

Outcome variable	Video-conferencing trolley, mean (SD)	HoloLens 2, mean (SD)	*p*-Value	95% CI	Effect size
Average ratings of usability (out of 5)^†^					
Rural Clinicians	4.75 (0.33)	4.45 (0.53)	.020*^§^	0.03–1.24	0.57
Advising Clinicians	4.25 (0.59)	4.69 (0.44)	.022*	0.11–1.41	0.77
Average ratings of self-efficacy (out of 5)^†^					
Rural Clinicians	4.27 (0.82)	4.43 (0.55)	.253	−0.23–0.89	0.33
Advising Clinicians	4.19 (0.56)	4.75 (0.45)	.008*	0.23–1.69	0.93
Average ratings of mental workload (out of 100)^‡^					
Rural Clinicians	25.52 (15.30)	30.38 (17.80)	.497^§^	−0.87–0.25	0.18
Advising Clinicians	35.75 (15.68)	27.63 (15.80)	.034*	0.05–2.32	0.70

*Note*: Analyses were performed with repeated-measures t-tests unless otherwise specified. Effect sizes are presented as Cohen's *d* for parametric tests and *r* (z/√N) for non-parametric tests.

^†^ Responses were collected on a five-point Likert scale with higher scores indicating better usability and more self-efficacy, respectively.

^‡^ Mental workload was scored out of 100 with higher scores representing higher mental workload (performance success item was reverse-scored to align with the other items).

^§^ Analysed with Wilcoxon Signed Rank Test due to violation of normality assumption according to a significant Shapiro–Wilk test.

* Significant at *α* < .05.

95% CI: 95% confidence intervals SD: standard deviation.

#### Usability

Advising Clinicians rated the HoloLens 2 higher on usability compared to the videoconferencing trolley. However, the results were flipped for Rural Clinicians, who rated the HoloLens 2 lower on usability compared to the videoconferencing trolley.

#### Self-efficacy

Advising Clinicians had higher ratings of self-efficacy when using the HoloLens 2 compared to the videoconferencing trolley. In contrast, Rural Clinicians rated their self-efficacy equally when using both devices.

#### Mental workload

Advising Clinicians reported that their mental workload was lower when using the HoloLens 2 compared to the videoconferencing trolley. However, for Rural Clinicians, perceived mental workload did not differ depending on the device.

### Secondary outcomes

#### Scenario duration

Using the video footage, scenario duration was calculated with scenario start and end times, subtracting any delays due to technical difficulties where the scenario was paused. On average, participants took 296 seconds (SD = 64) to complete the scenarios using the videoconferencing trolley, and 357 seconds (SD = 57) to complete the scenarios using the HoloLens 2, *p* < 0.001 (4:56 and 5:57 minutes, respectively). Table 4 presents the average scenario durations and associated *p* values to indicate statistically significant differences across devices.

**Table 4.** Descriptive statistics for average scenario duration across the two telehealth devices.

Average scenario duration (seconds)	Videoconferencing trolley, mean (SD)	HoloLens 2, mean (SD)	*p*-Value	95% CI	Effect size
Rural Clinicians	307 (62)	374 (60)	.033*	0.05–1.26	0.67
Advising Clinicians	283 (67)	337 (50)	.009*^§^	0.46–1.99	0.72

*Note*: Analyses were performed with repeated-measures t-tests unless otherwise specified. Effect sizes are presented as Cohen's d for parametric tests and r (z/√N) for non-parametric tests.

^§^ Analysed with Wilcoxon Signed Rank Test due to violation of normality assumption according to a significant Shapiro–Wilk test.

* Significant at *α* < .05.

#### Device preferences

**Figure 6.**
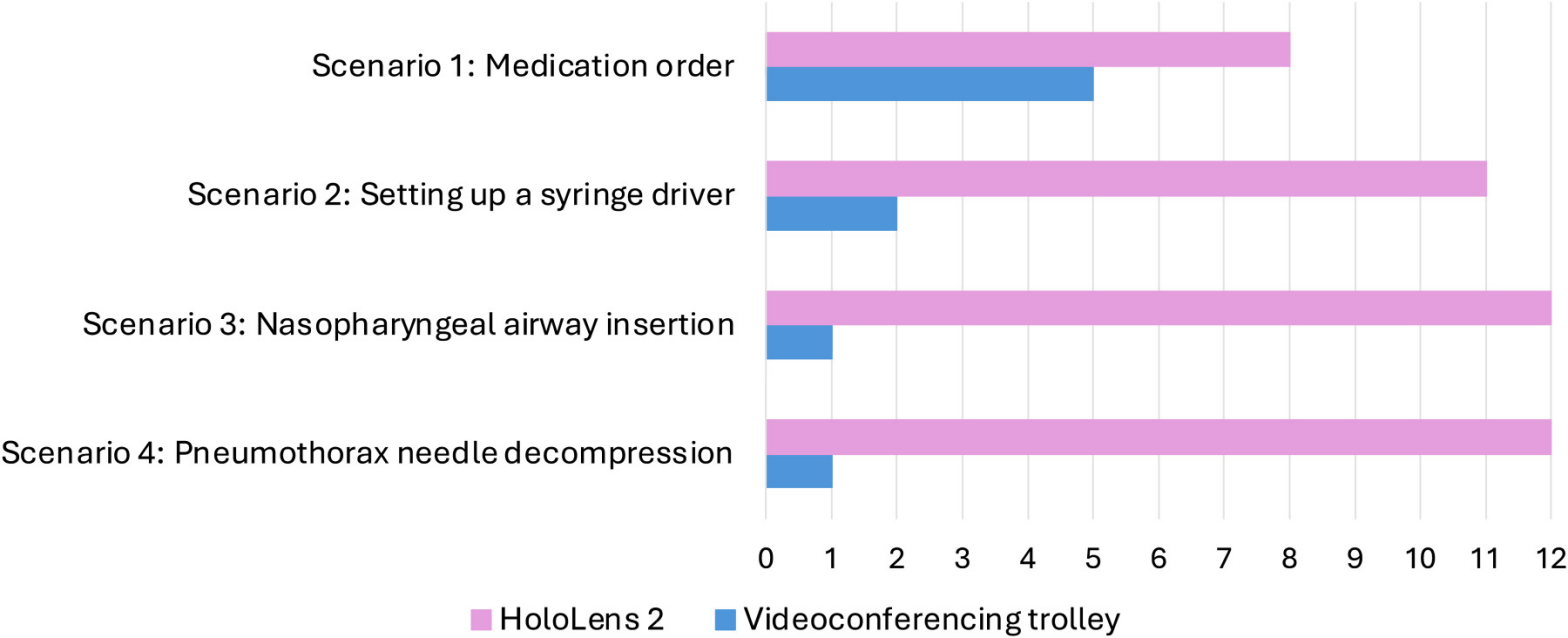
Rural Clinicians’ device rankings for each scenario.

**Figure 7.**
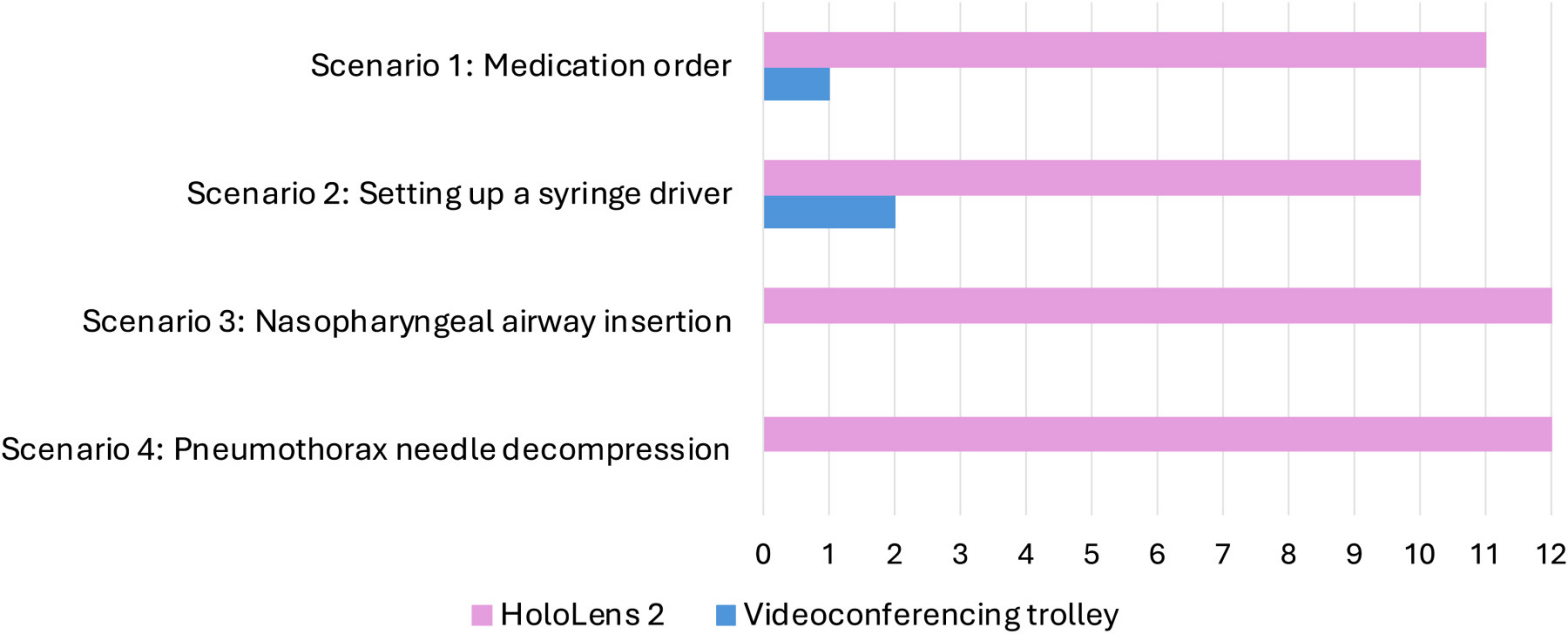
Advising Clinicians’ device rankings for each scenario.

Participants were asked which device they preferred to use for each scenario (Figures 6 and 7). Generally, both groups of participants preferred to use the HoloLens 2 over the videoconferencing trolley. However, scenarios 1 and 2 revealed the most variation, particularly amongst those in the Rural Clinician role.

#### HoloLens 2 feedback

Participants were asked to indicate how valuable they found four key functions of the HoloLens 2 (Table 5) on a 5-point Likert scale with 1 indicating no value at all and 5 indicating high value. Perceived value was high, with an average value rating of 4.79 out of 5 (SD = 0.27) for Rural Clinicians and 4.92 out of 5 (SD = 0.21) for Advising Clinicians.

**Table 5.** Perceived value of the HoloLens 2 key functions.

	First-person point-of-view camera	Annotations	Hands-free	Ability to share resources (e.g. documents, images)
Rural Clinicians				
Mean	4.77	4.62	4.92	4.85
Standard deviation	0.60	0.51	0.28	0.38
Percentage of participants who rated the function ≥4 out of 5	92%	100%	100%	100%
Advising Clinicians				
Mean	4.92	4.92	N/A	4.92
Standard deviation	0.29	0.29		0.29
Percentage of participants who rated the function ≥4 out of 5	100%	100%		100%

Responses were collected on a 5-point Likert scale with higher scores indicating higher value.

Participants were asked open-ended questions about the HoloLens 2's usability, comfort, visual and audio quality, likes, dislikes, sickness symptoms, and potential use-cases. A summary of these data are presented in Table 6.

**Table 6.** Participant responses on qualitative questionnaire items.

Qualitative questionnaire category	Rural Clinicians (n = 13)	Advising Clinicians (n = 12)
Usability and comfort (note: Advising Clinicians were not asked about comfort)	**Benefits:** Comfortable (9) Easy to use/intuitive (6) Own point-of-view available to Advising Clinician (5) Ability to have Advising Clinician ‘close by’ (4) Hands-free (3) Felt secure on the head / no readjustments needed (2) Ability to adjust the headset to fit own head (2) Ability to receive annotations (1) Ability to move objects in 3D space (1) **Challenges:** Technical difficulties (call drop outs, blurry annotations) (3) Less user-friendly with reading glasses (3) Navigating holographic interface with fingers (2) Had to tilt head position down to accommodate best view for Advising Clinician (2) Visor obstructed view just below eyeline (2) Fogged up when using a mask (2) Became uncomfortable over time (2) Unwanted pop-ups in 3D space (1) Clunky (1) Took time for visual perception to adjust (1) Took time to ‘get the hang of it’ (1) Activation of holographic interface overly sensitive (1) No control of camera angle or zoom (1)	**Benefits:** Ability to provide annotations and share files (8) Ability to view Rural Clinician's direct point-of-view (8) Easy to navigate interface/intuitive (7) Easy to communicate with Rural Clinician (2) Visual and audio clarity (2) **Challenges:** Could benefit from more practice interacting with system (6) Image quality (blurry, difficult to read up close) (2) Technical difficulties (call drop outs) (2) Had to ask Rural Clinician to tilt head position to accommodate best view (2) No control of camera angle or zoom (2) Rural clinician had difficulty finding Teams call in 3D space once connected) (1)
Visual and audio quality	**Positive experiences:** Audio quality (9) Clear and accurate annotations (8) Visual quality of holographic images (4) **Negative experiences:** Holographic images lack sharpness / a little faint (4) Annotations not pinning to the precise location in 3D space (2) Blurry annotations (2)	**Positive experiences:** Audio quality (8) Visual quality (4) **Negative experiences:** Challenging to read small text, e.g., on ampoule (2) Annotations not pinning to the precise location in 3D space (1) Slight delay in visual focus at times (1)
Perceived benefits in clinical care	Own point-of-view available to Advising Clinician (9) Could improve confidence in clinical care (e.g. for rural and remote clinicians, inexperienced clinicians) (6) Hands-free telehealth device (5) Able to spend more time at the bedside (3) Ability to view X-rays directly at bedside (2) Feels like the Advising Clinician is physically present (1) Could improve patient confidentiality (1) Could reduce travel requirements for rural and remote patients (1)	Ability to view Rural Clinician's direct point-of-view (8) Could enable safer patient care (e.g. in rural and remote areas) (6) Ability to provide annotations and share files (4) Feels physically present with the Rural Clinician (3) Could improve confidence for Advising Clinicians (2)
Perceived concerns in clinical care	Usability and comfort (e.g. downward neck tilt required) (6) Mixed Reality functionality may not be ‘ready’ for clinical use (3) Time required to become familiar with device (2) Potential for sickness symptoms with prolonged use (2) Technical issues could delay care (1) Could slow down care in emergent situations (1) Patient not able to see Advising Clinician (1) May affect maintenance of eye contact with patient (1)	Restricted field-of-view (having to ask Rural Clinician to tilt head position to accommodate best view) (4) Technical issues could delay care (4) Time required to become familiar with device (2) Potential for sickness symptoms with prolonged use (2) Precision may be compromised with annotations (1)
Sickness symptoms	Mild headache (2) Minor blurred vision (2) Slight dizziness (1) Nausea (1)	Potential to experience symptoms with prolonged use (2) Disorientating (2)
Potential clinical use cases	**Useful or appropriate for:** Complex interventional and emergent procedures (intubation, airway manoeuvres, etc.) (11) Non-emergency cases (patient assessment, midwifery care) (3) Tasks where both hands are required (2) Education and training (1) **Not useful or appropriate for:** Patient interactions/consultations (3) Patients with behavioural or mental health issues (3) Emergency cases (2) Where patient modesty is required (1) Child or teenage patients (1) Back-to-back consultations (due to potential discomfort with prolonged use) (1) Cases where multiple team members are involved in telehealth consultation (1) Palpation assessments (1)	**Useful or appropriate for:** Patient assessment (5) Bedside procedures (4) Emergency cases (4) Education and training (3) Novel / unfamiliar tasks (2) Patient retrievals / prehospital (2) Equipment troubleshooting or demonstration (2) Wound care (1) Neonatal resuscitation (1) Non-invasive monitoring (1) Subacute cases (1) Cases where varying views or angles are important (1) Prenatal care (1) Aged care (1) Paediatric care (1) **Not useful or appropriate for:** Cases where multiple team members are involved in telehealth consultation (3) Rural and remote locations with poor internet connectivity (2) Emergency cases (3) Team-based care (1) Technical / interventional procedures requiring precision (1) Cases requiring cultural sensitivity (1) Cases requiring basic guidance (1) Child or teenage patients (1) Sensitive examinations (1)

Corresponding values indicate the number of participants who provided feedback.

#### Scenarios

Participants had varying degrees of experience with the scenarios chosen for this study. Thirty-eight percent of participants had experience with all scenarios used in this study, and all remaining participants had experience with at least one of the scenarios. On average, participants rated the scenarios 4.52 out of 5 (SD = 0.65) on realism and 4.44 out of 5 (SD = 0.71) on immersion. When asked if they behaved the same as they would in authentic clinical situations, participants rated the scenarios 3.96 out of 5 (SD = 1.06).

## Discussion

### Summary of findings

The aim of this study was to investigate the usability of a MR headset for clinician-to-clinician telehealth in rural patient care and compare it to a videoconferencing device available in current practice. The findings suggest that telehealth via the HoloLens 2 has many advantages over traditional videoconferencing, and most participants in both groups preferred to use the HoloLens 2 over the videoconferencing trolley in all scenarios. These findings are consistent with recent literature highlighting the potential of MR to enhance clinical communication and procedural support.^5,22^ This is promising for application in rural areas, where geographic distance creates challenges in providing equitable healthcare.^1,23^

However, the HoloLens 2 may benefit those dialling in to it (advising clinicians) more than those wearing it (rural clinicians). This finding is consistent with a previous usability evaluation highlighting the differing requirements of each end-user group.^24^ For participants in the Advising Clinician role, the HoloLens 2 elicited higher ratings of usability and self-efficacy, and lower ratings of mental workload, compared to the videoconferencing trolley. However, participants in the Rural Clinician role had less consistent results. There were no differences in ratings of self-efficacy and mental workload across the two devices, but ratings of usability were lower for the HoloLens 2 compared to the videoconferencing trolley. From a Rural Clinician perspective, the videoconferencing trolley is simple to use because once a call is connected, they become a passive user. The HoloLens 2 requires more active participation through hand gestures that may be difficult to grasp for a first-time user. Through increased exposure, participants may become more familiar with the headset and find it more usable.

When participants used the HoloLens 2, scenarios took one minute longer on average than when they used the videoconferencing trolley. This effect remained when the two participant groups were analysed separately. Two scenarios involved annotation and file sharing which is probably what increased the scenario durations. However, these functions, along with the first-person point-of-view, were rated as highly valuable for both participant groups. Although not directly tested, these functions would likely support improved procedural accuracy. Therefore, the speed-accuracy trade-off of 1 minute may be acceptable in many clinical situations. Other studies have found similar trends, where MR use elicits longer completion times while simultaneously being reported as more beneficial than existing options.^25,26^ Importantly, the time taken to resolve HoloLens 2 technical issues was removed from the overall scenario duration. This was done to ensure that the duration analysis was assessing whether the HoloLens 2's functionality slowed down or sped up clinical tasks compared to the videoconferencing trolley. The most common technical issue was the holographic Teams call appearing in an undesirable location in 3D space, becoming unmovable, or disappearing altogether. These issues required restarting the call or headset. Clinical care could be delayed if these issues occurred in real clinical practice.

Qualitative feedback revealed perceived benefits and limitations of the HoloLens 2 for each end-user group. Participants in the Rural Clinician role found it easy to use and useful to communicate via a hands-free device; however, navigation of the holographic interface was not instantly intuitive. Participants generally found the HoloLens 2 comfortable and light to wear. Because the camera was positioned higher than eyeline, participants typically had to tilt their head downwards slightly so the Advising Clinician could see their field-of-view. Holding this head position may become uncomfortable over time. The holographic interface and restricted field-of-view are common criticisms of the HoloLens 2.^27,28^ The efficiency of having the Advising Clinician ‘nearby’, and directly viewing their point-of-view, was of significant benefit. Participants perceived the visual and audio quality to be high, but in some cases the annotations were blurry, faint, or not pinned to the precise position. Although the headset is compatible with glasses, some participants experienced difficulties wearing both simultaneously. Minimal sickness symptoms were experienced for the wearers, except for one participant who experienced significant nausea.

Participants acting in the Advising Clinician role found providing guidance via the HoloLens 2 relatively easy once familiar with it. Participants found it easier and more efficient to give instructions using the first-person point-of-view paired with the ability to annotate and share files in real time. Rather than relying solely on verbal instructions and subsequent interpretation of those instructions, Advising Clinicians could easily confirm that their instructions were being followed and that information was accurate. However, sometimes the video did not focus instantly which increased the time needed to read details (e.g. drug labels). There were minimal issues using Microsoft Teams, perhaps due to ubiquitous use across the state. As previously noted, there is discrepancy between the camera field-of-view and the wearer field-of view, so Advising Clinicians had to instruct participants to tilt their head down at times. As a result, there was a desire to have some degree of remote camera control.

Participants suggested potential use cases for the HoloLens 2, such as technical procedures and patient assessments. However, participants also highlighted potentially ill-suited applications, including patient consultations and situations requiring multiple team members’ involvement in a telehealth consultation. Interestingly, emergency care was suggested as both a situation that the HoloLens 2 would, and would not, be appropriate for.

### Practical implications, limitations, and future directions

This study was conducted in Queensland, Australia, which is the country's second largest state. With an area exceeding 1.7 million square kilometres and nearly 40% of people living rurally,^29,30^ Queensland records one of Australia's highest usages of telehealth services,^31^ including dedicated infrastructure that facilitates 24/7 communication between rural clinicians and metropolitan specialist clinicians.^1,32^ Although clinician-to-clinician telehealth is viewed as a critical service that supports rural clinicians and benefits patient care, there have been recent calls for improvement including increased portability and the ability to exchange information more easily.^1^ Wearable MR videoconferencing shows promise to complement the existing Queensland telehealth infrastructure and address some of these existing shortfalls. However, our findings highlight the need for further investigation to mitigate safety and efficiency concerns for both end-user groups.

This study had several limitations that could affect generalisability. First, not all participants in the Rural Clinician condition had experience working rurally. This was due to practical constraints given the face-to-face nature of the study. Second, this was a low-fidelity simulation study which highly simplified the clinical context. It is likely that ratings of usability and mental workload will be different in an emergency telehealth scenario. Third, telehealth experience was not measured or accounted for, which could influence perceptions of both existing technology and new technology. Fourth, clinical performance was not directly measured (although indirectly measured through usability, mental workload, and self-efficacy^14–16^) so the impact of the HoloLens 2 on performance or patient outcomes remains unknown. Fifth, data collection occurred between blocks, rather than between scenarios. This was intentional to allow participants enough time to become familiar with each device, and because the focus was on device usability and feasibility across a range of scenarios. Finally, both the Advising Clinician and Rural Clinician were stationed in adjacent rooms using the same internet connection. Device use in rural areas will often experience poor internet connectivity. These limitations should be investigated in future work to guide successful implementation of novel telehealth technologies in clinical practice. For example, a clinical trial of the HoloLens 2 would be a crucial step prior to implementation to measure the effect on clinical performance and patient outcomes, and to test the technology across varying internet quality over vast distances. In addition, MR headsets may not be the best solution for critical emergency and trauma care, and may be better suited to lower acuity situations. A set of recommended use cases and conditions for wearable MR videoconferencing should be developed so that it is only used when and where it will provide the most benefit.

It should also be noted that as of October 2024, Microsoft discontinued the sale and development of the HoloLens 2, and no successor device has been announced. The device is no longer available for purchase, but existing devices will be supported until 31 December 2027. The present study was conducted several months prior to the announcement of the discontinuation. Regardless, the findings can be generalised to other comparable devices on the market and can inform the design of future wearable MR devices. Future studies on wearable MR telehealth will require new market scanning for appropriate technologies and devices available at that time which meet clinical requirements.

## Conclusions

As we progress toward more advanced telehealth models of care, there is an increasing need for new features and technologies. This simulation-based usability study provided crucial insight into the benefits and limitations of a MR headset for telehealth. The findings suggest that wearable MR videoconferencing has many advantages over traditional videoconferencing; however, the HoloLens 2 provides a different experience depending on whether a clinician uses it to provide support or access support, and it may benefit the former more than the latter. Wearable MR may not provide value over traditional videoconferencing in certain situations and may even cause disruption in others. Overall, the results suggest that wearable MR videoconferencing has the potential to improve support for rural clinicians, but there are various factors that, if not adequately considered, may hinder implementation.
